# The properties of panels in global comics: frequency and size of 76 K panels in 1,030 comics from 144 countries

**DOI:** 10.1007/s10579-026-09925-9

**Published:** 2026-05-26

**Authors:** Neil Cohn, Andrew T. Hendrickson, Bruno Cardoso, Bien Klomberg, Irmak Hacımusaoğlu, Ana Krajinović, Sharitha van der Gouw, Fred Atilla, Abe Simons, Tim Hankart, Nanne van Noord, Sam Titarsolej, Fernando Casanova Martínez, Tomás Gaete Altamirano, Marianna Pagkratidou, Michał Szawerna, Nicolas Verstappen, Heinz Insu Fenkl, Leandro Kruszielski, Anna Marta Marini, Gaurav Singh, Dušan Stamenković, Miloš Tasić, Yen Na Yum

**Affiliations:** 1 Department of Communication and Cognition, Tilburg University, Tilburg, Netherlands; 2 Department of Cognitive Science and Artificial Intelligence, Tilburg University, Tilburg, Netherlands; 3 Informatics Institute, University of Amsterdam, Amsterdam, Netherlands; 4 Department of Spanish Philology, General Linguistics and Theory of Literature, University of Alicante, Alicante, Spain; 5 Department of Psychology, Universidad Autónoma de Chile, Santiago, Chile; 6 Faculty of Science and Health, School of Psychology, Dublin City University, Dublin, Ireland; 7 Institute of English Studies, University of Wrocław, Wrocław, Poland; 8 Faculty of Communication Arts, Chulalongkorn University, Bangkok, Thailand; 9 Department of English, State University of New York, New Paltz, USA; 10 Department of Theory and Foundations of Education, Federal University of Paraná, Curitiba, Brazil; 11 Instituto Franklin, Universidad de Alcalá, Madrid, Spain; 12 Independent Researcher, Lucknow, India; 13 Faculty of Philosophy, University of Niš, Niš, Serbia; 14 Faculty of Mechanical Engineering, University of Niš, Niš, Serbia; 15 Department of Special Education and Counselling, The Education University of Hong Kong, Tai Po, Hong Kong; 16 Department of Information Technologies, Institute of Technology, Universidad CEU San Pablo, Madrid, Spain; 17 Department of German Studies and Linguistics, Humboldt University of Berlin, Berlin, Germany

**Keywords:** Visual language, Comics, Corpus analysis, Panels, Menzerath–Altmann law, Linguistic relativity

## Abstract

Panels are a fundamental unit of comics, yet basic data about their usage in comics from around the world has not been widely investigated. Here we analyze panel information in the TINTIN Corpus consisting of 1,030 comics from 144 countries—comprising over 14,000 pages with over 76,000 panels—all annotated using the Multimodal Annotation Software Tool (MAST). We examined both the number of panels per page and the relative size of those panels to their pages, finding that they varied in dimensions of the style that they are drawn in, the global region they come from, the typological properties of the languages spoken by their authors, and the year of their publication. In addition, a clear tradeoff occurred between these dimensions of structure, where larger panels appeared for fewer panels per page, and vice versa. This relationship appeared to be “universal”, persisting similarly no matter the variation across style, region, language, or publication date of the comics. Altogether, this work reveals that the structure of panels on comic pages involve a tension between variability across numerous sources and universal consistencies of properties that persist across all comics.

## Introduction

**Fig. 1:**
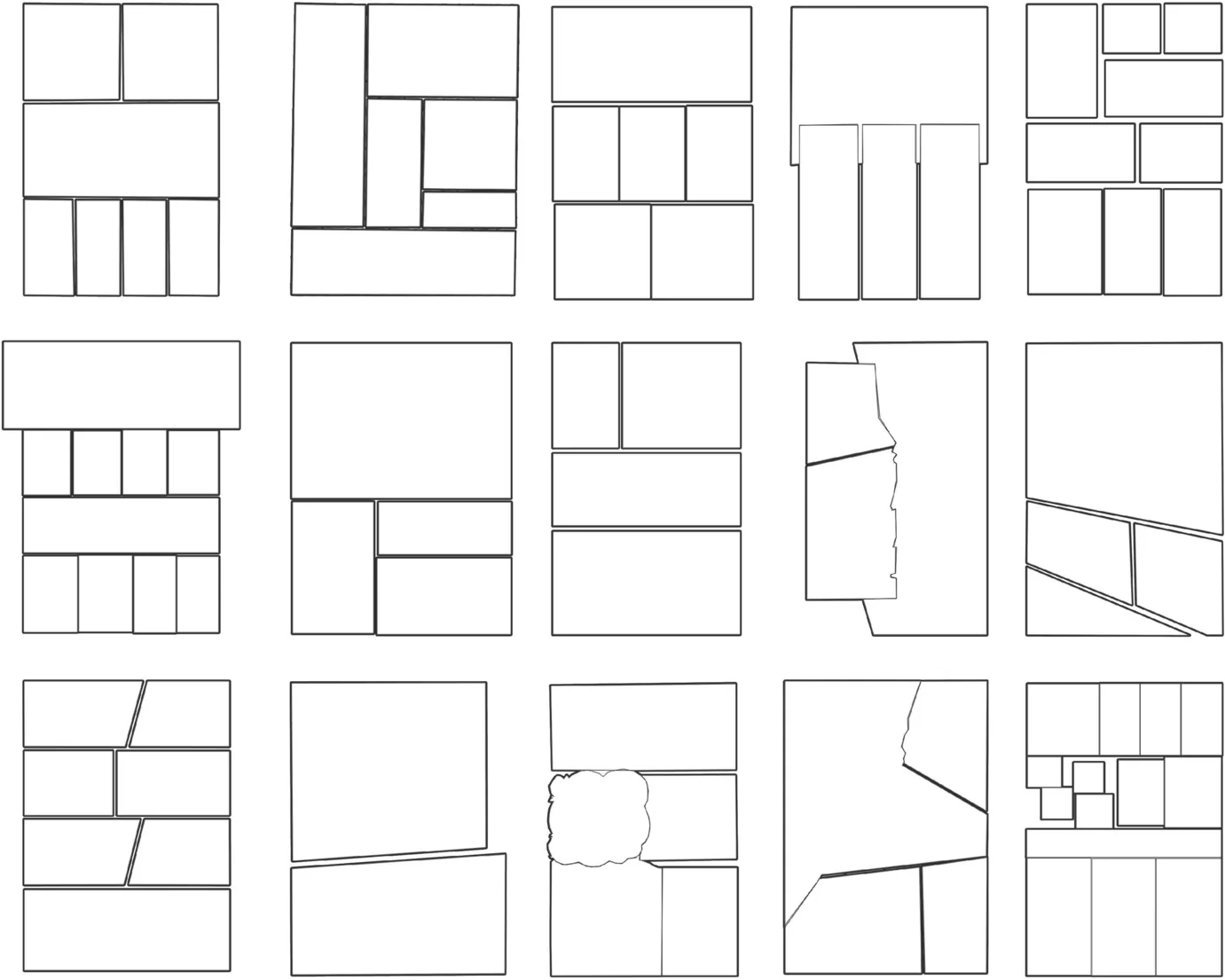
Fifteen page layouts from the 14,324 pages from 1030 comics in the TINTIN Corpus

Panels are often described as one of the fundamental units of comics (Duncan et al., 2015; Groensteen, 2007; McCloud, 1993), and they mark layouts “as comics” even in the absence of content, as in Fig. 1. These encapsulating graphic spaces function across multiple substructures of the visual languages used in comics, serving as units of layout and storytelling while also maintaining their own internal composition in relation to surrounding panels (Cohn, 2024). Yet, despite their primary functions in visual narratives, little empirical work has detailed the basic properties of panels in actual comics, and especially not with a global scope. This paper therefore provides the most extensive analysis of comic panels to date, asking: what factors go into the frequency and size of the panels on comic pages?

Empirical studies of comics have been growing over the past two decades, both with those using computer vision techniques to automatically extract features of comics, often from computer science (Augereau et al., 2018; Laubrock & Dunst, 2020), and those constructed using manual annotations, often from the language and cognitive sciences (Bateman et al., 2021; Cohn, 2024; Dunst et al., 2017). Participants have high agreement in the annotation of panels (Edlin & Reiss, 2021), and correspondingly segmentation of panels from pages using computational methods has become quite reliable, often reaching consistently high accuracy in selecting and identifying panels (Ikuta et al., 2023; Sharma & Kukreja, 2024; Zhang & Hotta, 2023). However, this automatic extraction has largely not been used as an analytical tool for studying the properties of comics more broadly.

Corpus analyses that look at the content of comics’ structures have surveyed a range of topics, including, but not limited to, how layouts have changed over time (Bateman et al., 2021; Cohn, 2024), combinations of stylometric features (Dunst, 2023; Laubrock & Dubray, 2019), aspects of storytelling (Bateman et al., 2021; Cohn, 2024; Iyyer et al., 2017), bibliographic data (Beaty et al., 2018; Dunst, 2021, 2023; Dunst et al., 2017), or genres (Xu et al., 2023). Despite this growing proliferation of corpus research, most works have reported minimal information about the basic distribution of panels on pages, or information about their relative sizes or shapes. In addition, most corpora have been constrained in scope to characterizing comics within or between particular countries with prominent comics industries (Japanese manga, American comics, Franco-Belgian bande desinée), types (manga, graphic novels), or genres (independent comics, superhero comics).

A broader scope is found in the Visual Language Research Corpus or VLRC (Cohn et al., 2023), which includes annotations of 48,485 panels from 361 stories in comics from the United States, Asia (Japan, Korea, China), and Europe (Belgium, France, Germany, Netherlands, Sweden). Several countries also included distinctions between subtypes of comics, such as contrasts in American comics between mainstream, independent, and US manga. Also, while all countries had data from the 1980s to 2010s, those from the United States, The Netherlands, and Flemish Belgium also extended back to the 1940s. While it was not balanced across the corpus, this heterogeneity provided a range of ways to characterize comics of widely different types, including their basic paneling.

Analysis of panels within the VLRC showed that on average, pages had 6.4 panels per page, with standard deviation of 2.6 and a range of 1–24 panels (Cohn, 2024). However, European comics had the highest quantities of panels per page, with an average of 7.94 panels per page, particularly from comics from Flemish Belgium with an average of 10.4 panels per page. By contrast, fewer panels per page were used in comics from the United States (5.3) and Asia (5.0). In addition, these frequencies had changed over time, with comics in the United States reducing in the average number of panels per page, from 6.2 in the 1940s to 4.8 in the 2010s. European comics changed less consistently, starting at around 9.2 panels per page in the 1940s, and then oscillating between 7 and 9 panels per page across the decades. The VLRC has no datapoints for Asian comics before the 1980s, but panels per page hovered consistently around 5 from the 1980s to 2010s for books from Korea, Japan, and China.

Nevertheless, the data in the VLRC is also limited. Despite its cross-cultural scope, it included data from only 9 countries across North America, Europe, and East Asia. Of these, most of the comics came from the 2000s, while only those from the United States, the Netherlands, and Flemish Belgium extended with publication dates to the 1940s. In addition, the VLRC data was gathered solely through spreadsheets, often analyzing physical copies of books. This meant that no assessment could be made of the actual properties of the physical pages themselves.

To better investigate aspects of the structure used in comics, in this case panels, the TINTIN Project has worked to establish a corpus of annotated comics with a more global scope. The TINTIN Corpus consists of 1,030 comics from 144 countries or territories spanning all continents of the globe (except Antarctica). These comics have been annotated using the Multimodal Annotation Software Tool, or MAST (Cardoso & Cohn, 2022), which facilitates better understanding of the size and relationships between aspects of the structures used in multimodal documents like comics. This software allows for the selection of flexibly shaped regions of interests in a document which can then be annotated with any number of user-specified classifications (Cohn et al., 2024). In this paper, we target the frequency, measured in panels per page, and size of panels to gain a better understanding of the basic units used in comics.

There are various dimensions by which panels might vary systematically across comics. A first hypothesis might be that we observe no substantive variation across comics of the world at all. Such a finding would support a view that there is merely one persisting “comics medium” across all comics, and any variation observed between them should thereby be incidental. This position is often maintained both explicitly or implicitly by many approaches to the study of comics (e.g., Duncan et al., 2015; Exner, 2021; Gavaler, 2022).

If such consistency is not observed, a first dimension of variance might be cross-cultural. Here we might expect comics from different parts of the world to vary by virtue of constraints by cultural practices and/or the publishing practices in different regions of the globe. Indeed, hints of cultural differences in comics’ structure have been observed across dimensions like layout and panel framing (Cohn, 2024), backgrounds (Atilla et al., 2023), among others. Related to paneling, different cultural publishing traditions may affect the number of panels per page through their standardized formatting. For example, Franco-Belgian albums have physically larger pages, and thus more panels (Cohn, 2024), while the absolute size of comic books or manga is comparatively smaller, and thus presumably with fewer panels (Lefèvre, 2000).

Nevertheless, other structures have been observed to cut across cultural boundaries (Cohn, 2024; Cohn et al., 2026; Hacımusaoğlu & Cohn, 2022), and some prior observations of “cultural” distinctions may be confounded with other dimensions, such as the “style” used by those comics. Indeed, previous analyses of comic panels in the VLRC showed that panels in comics from the United States and Asia were more similar compared to those from Europe, suggesting cultural boundaries were not defining these tendencies (Cohn, 2024).

An alternative way that comics may vary is based on their particular “visual language,” i.e., the systematic structures shared by creators resulting in distinctive graphic systems (Cohn, 2013, 2024). One of the aims of the TINTIN Project is to seek whether it can find empirical evidence substantiating different visual languages persisting across comics, and ultimately these classifications are sought to be examined “bottom up” via the annotation data itself, as in analyses of the VLRC (Cohn, 2024). Because of this, we did not directly assign comics to visual languages but instead used a proxy for distinguishing visual languages by their visual “styles” (see Methods), such as the graphic differences based on the linework of how American superhero comics are drawn relative to how Japanese manga are drawn. The drawing style reflects the “lexicon” of a visual language, which joins other structures guiding all facets of how graphic systems communicate (i.e., layout, storytelling, etc.).

If panels do support the presence of visual languages, we might therefore expect patterning to cut across cultural dimensions in line with the proxy of their “styles”—superhero or manga styled comics should thus pattern together, no matter their global or cultural origins. Indeed, though languages are often associated with particular countries of origin, their spread does not respect geographic boundaries, such as how due to colonialism and media influence English (or English*es*) is spoken across the whole world, not just bound to England. Some analyses of the VLRC already implied this, where many works of “manga” created in North America also clustered in their features with manga from Japan, manhua from China, and manhwa from Korea (Cohn, 2024). In other analyses of the TINTIN Corpus, styles make for better predictors than global regions, and/or styles maintain consistent properties across global regions for structures like layout (Cohn et al., 2025a, 2025b), flow (Cohn et al., 2025b), and framing (Cohn et al., 2026). This has suggested that these structural features were more “cross-linguistic” than cross-cultural and provide evidence for visual languages that cut across cultural boundaries. To this end, it remains an open question whether the distribution of panels is one such structure that is indicative of such variation, or whether panels vary (or not) for other reasons.

The distribution of panels in pages may also vary based on the publication date of the comics. As described above, analyses of the VLRC suggested that the number of panels per page decreased from around 6.2 to 4.8 in comics from the United States, while less consistent trends were displayed by Dutch and Flemish comics over time (Cohn, 2024). Given this precedent, we might similarly predict a decrease in the number of panels per page across publication dates in comics from more global origins. Changes in publication date may also align with differences that occurred given changes in format or publishing practices, such as a shift in the physical size of comic pages or the length of pages in a book, given specific industries tied to cultural contexts (Lefèvre, 2000). Nevertheless, consistent global changes in panels might suggest broader shifts that transcend such national or cultural industries.

Another possibility is that panels vary across dimensions of the languages spoken by their creators. In some prior work, dimensions of spoken language structure have been shown to affect the visual representation of motion events (Hacımusaoğlu & Cohn, 2022; Tversky & Chow, 2017), and the complexity of panels’ framing and narrative structures (Cohn, 2024). There is thus the possibility that typological aspects of linguistic structure may also affect the distribution of panels.

In this case, we considered panels per page as a type of meaningful segmental structure, with a closest connection to the segmentation of relative density of morphemes in words (Bickel & Nichols, 2013; Haspelmath, 2017; Koplenig et al., 2017). To be clear, we are *not* directly equating panels on a comic page with morphemes in a word. Rather, the reasoning here would be that a general orientation for segmental complexity might *permeate* between modalities (Cohn, 2024), such that the complexity of morphemes in a word might habituate readers towards a broader inclination for the ways they segment other meaningful expressions, such as panels on a page. This would follow similar reasoning to effects of general typological predictions of phonological tone on musicality (Liu et al., 2023) or syntactic structure on working memory (Amici et al., 2019), only here with segmental preferences of morphological structure reflected across units of a page.

To summarize, we posited that panels per page may have potential sources of variation from the global or cultural origin of the comics (here analyzed via geographic region), their visual language (here analyzed via the proxy of style), their publication date, or possibly the typological properties of the language of their authors. We explore these factors below both for the number of panels per page and the size of panels.

Finally, like in spoken and signed languages we considered the possibility that, despite variation occurring across systems, regularities may span across varieties in ways consistent with being linguistic universals. We not only predict such that such universals would span across all types of comics, but would be consistent with well-established universals pervading spoken and signed languages (Cohn, 2024; Cohn et al., 2026; Krajinović et al., 2025). In this case, we predicted a negative relationship between the frequency of panels on a page and the size of those panels—i.e., more panels per page should lead to smaller panels, and fewer panels should lead to bigger panels. A canvas has a potentially infinite number of ways it can be filled that do not mandate completely occupying that space. If a finite space is filled entirely, then increasing numbers of panels will become smaller, but it is also possible to have just a few small panels on a large page, “free floating” panels, and/or large gutters. So, a trade-off between frequency and size would be the logical expectation, but it is not inevitable given the range of possibilities. If such a relationship is maintained, it would uphold the Menzerath–Altmann Law (Altmann, 1980; Menzerath, 1928), that the larger a construct, the smaller the size of its constituents.

## Methods

### Materials

The TINTIN Corpus consists of 1,030 annotated comics from around the world. The primary aim of the construction of the TINTIN Corpus was to achieve *global coverage* of comics from as many representative countries as possible. This was achieved through convenience sampling, acquiring comics through direct donations of comics from over 80 comic creators and publishers, from contributions from collaborative scholars in various countries, and through public domain comics on the Internet. The corpus includes comics published by professional publishers (mostly tied to different countries), those published by digital publishers (with more global reach), and those created entirely by amateurs with no intent on published distribution.

To this extent, it is worth emphasizing that the TINTIN Corpus does not aim to study the properties of “comics” as published objects of social institutions (cf., Dunst et al., 2017), but rather to study the properties of the expressive systems used in comics worldwide—i.e., the visual languages of their authors—regardless of professional publication status. For this reason, the corpus does not emphasize particular countries with industrial publishing histories, specific authors or publishers, or particular “canonical” comics. Rather, it seeks a broad sampling of graphic narratives that can allow for the identification of regularities and sources of variance based on the structural properties of the materials.

Because of the disparate nature of this sampling and the resources available, it created a heterogenous distribution of comics within each country and global region regarding publication date, style, genre, and other distinctions. In some cases, the rich traditions of comics in countries led to simple sampling of a range of comics to include, while other cases comics were difficult to find and we only managed to find a few representative titles from certain countries. Nevertheless, the heterogeneity of the corpus is in part intentional, as existing research has not made it clear which aspects of comics lead to systematic variance (if theories posit such variance or systematicity at all), or make assumptions about diversity based on socio-cultural and/or theoretical distinctions that have not been empirically verified. Thus, the wide diversity throughout the corpus can hopefully allow for research to disentangle the meaningful properties of comics’ classifications.

**Fig. 2:**
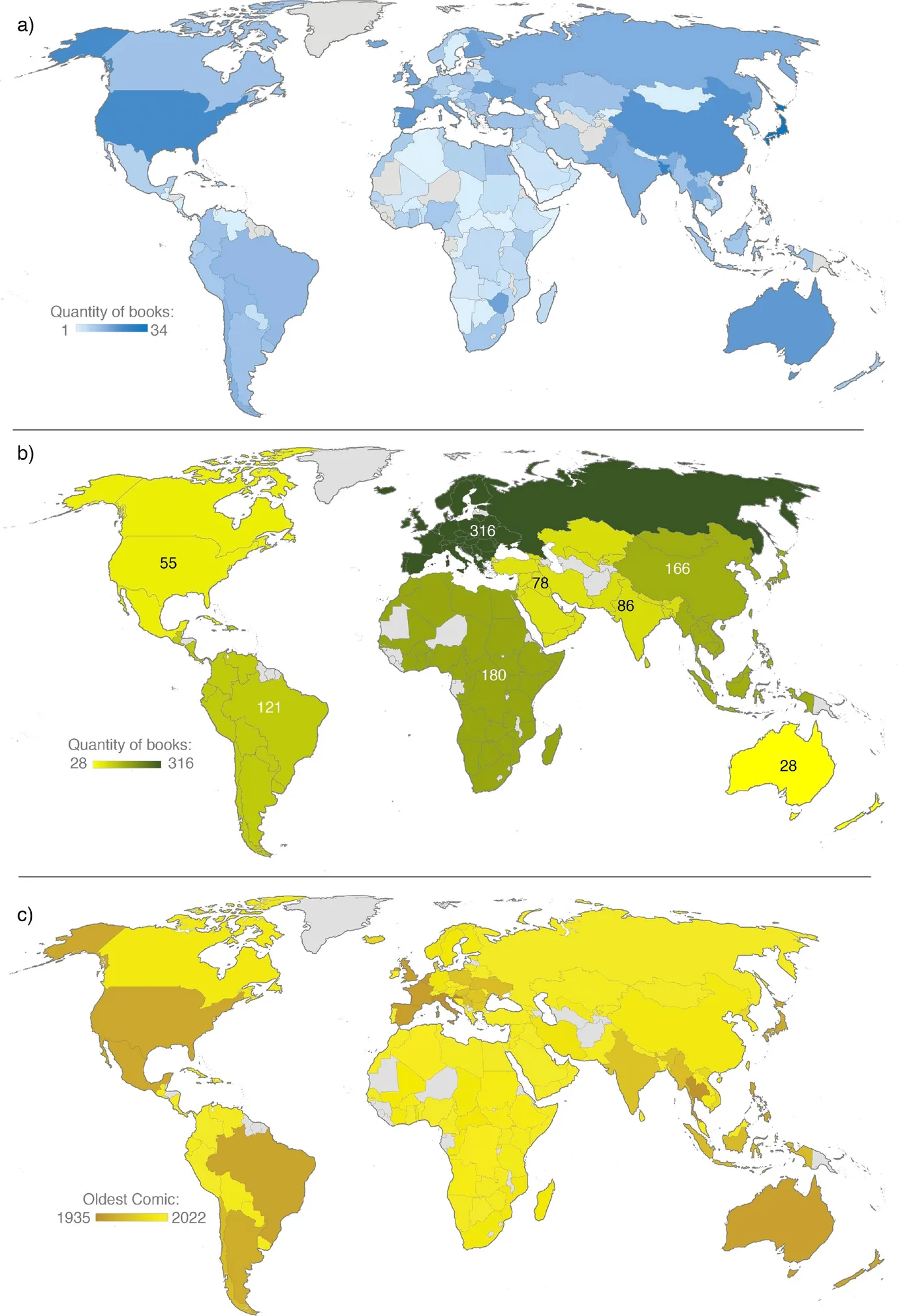
The makeup of the TINTIN Corpus in terms of **a** Distribution of comics within each country (darker blue means more comics in the corpus), **b** The distribution of countries into each global region, and **c** The publication date of the oldest comic in the corpus in each country (darker means older)

Nevertheless, global coverage within the TINTIN Corpus was achieved with 144 distinctive countries or territories represented across all continents of the world (except Antarctica), as depicted in Fig. 2a depicting the distribution of comics across a map of the world. Our assigned global regions for countries appears in Fig. 2b. The oldest comic was from 1935 and the newest was from 2023, with most (82% 841/1,030) published between 2000 and 2023. Figure 2c maps the oldest publication date of comics in the corpus from each country. All recorded properties of the comics in the corpus along with vector-based recreations of all page layouts of the corpus can be found in its DataverseNL repository: https://doi.org/10.34894/KSVSRL.

### Style

A second important property of the comics within this corpus is their designation of “style,” which is a classification of the visual “drawing style” of the comics. Within the TINTIN Project, the classification of visual languages is sought as an empirical question, and thus “style” is used as a proxy for different visual languages, since the linework reflected by different styles designates the “visual vocabulary” of different systems. Nevertheless, individual visual languages can potentially use multiple types of graphic styles, and there may be typological similarities and variance across different visual languages. In addition, though stereotypical or idealized styles may be identifiable, it may also exist on a continuous dimension reflecting the idiolects of various authors. Our stylistic dimensions here thus aim to capture variability that may be indicative of these different types of representational systems, and our methods aimed to provide both a discrete and continuous classification of styles.

We used a combination of manual annotation and computer vision methods. We first manually annotated drawings styles with diverse labels by visual inspection of the graphic representations of the comics. Each comic was assigned a style by at least two annotators. These labels were then assessed and revised using computer vision methods (below).

Our computational assessment of style was based on analysis of panels within their sequence (for details, see Titarsolej et al., 2024). We used self-supervised learning techniques commonly used in masked language modelling (Devlin et al., 2018) here applied to sequences of comic panels through a transformer encoder (Vaswani et al., 2017). Comics were first greyscaled to eliminate influence of color, because color “distracts” the model into grouping color pages as being more similar to other color pages, regardless of additional information. For example, comics with linework in a manga style would be expected to be fairly similar, but if one was in color and the other in black and white, the model might render them distinct due to the color alone, and/or group the color manga with a color superhero style rather than assessing their linework.

Next, the coordinates for panel information (see below) on pages were used for an encoder to compare panels across the corpus. The encoding model was trained with a self-supervised task using all the panels from the TINTIN Corpus. A panel encoder was trained on a sequence of panels by optimizing it to predict a single masked panel to learn to extract meaningful information from the other panels in a sequence in relation to this masked panel. The panel sequence encoder used a Vision Transformer (ViT) (Dosovitskiy et al., 2020) pre-trained using DINO (Caron et al., 2021), which can capture various panel-level context aspects such as coarse style. Rather than predicting pixel values of the panel, which can lead to small differences negatively influencing training, the model was trained to retrieve the correct panel based on a pool of candidate panels including the correct panel and several distractors. A second task gave the model sequences of panels in a random order, from which it needed to determine the correct order. This task optimized the model for longer distance dependencies between panels.

**Fig. 3:**
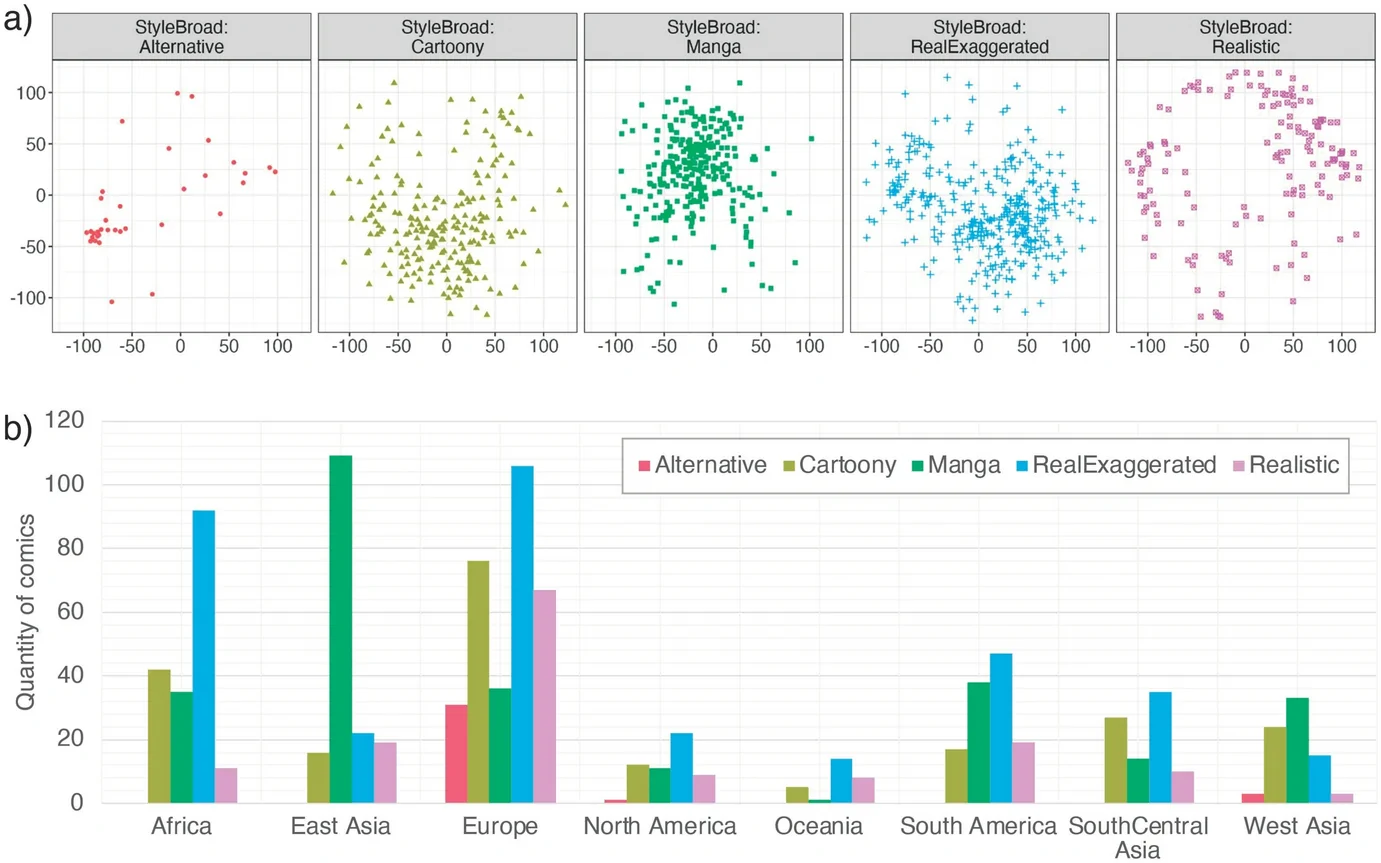
Stylistic dimensions of the comics in the TINTIN Corpus shown through **a** a t-SNE plot for the computationally derived analysis values for panel similarities averaged per comic in a relational way, and **b** number of comics for each style within each global region

A final step performed dimensionality reduction on the extracted panel features using t-SNE (Van der Maaten & Hinton, 2008) to reduce the high-dimensional encodings to two dimensions which preserves relative distances between vector points (i.e. panels). The t-SNE plot shows a projection of the data in a synthetic two-dimensional coordinate space, with points closer together in 2D also closer together in the original high dimensional space (Fig. 3a). To assess the styles of whole comics, we then averaged across the t-SNE values for a given comic and compared how the manually annotated styles of comics manifested within the vector space of t-SNE values. We grouped together styles that appeared to overlap in their t-SNE vectors and sought to arrive at stylistic classifications with the most distinct t-SNE mapping that also aligned with the manual designations.

This process arrived at several levels of classification for styles, though ultimately describing a continuous dimension of style that can be further classified and/or have other structures mapped into it (Fig. 3a). Here we focus on our broadest stylistic dimensions, with five semi-distinct style clusters: Manga, Cartoony, Real-Exaggerated, Realistic, and Alternative. *Manga* characterized the style stereotypical of comics originating from Japan, which has subsequently extended across the global manga (Brienza, 2015) in our corpus. *Cartoony* characterized comics with exaggerated and plastic features without proportions maintaining a sense of accuracy with real human figures. *Real-Exaggerated* characterized comics with exaggerated features, but with figures more maintaining real proportions, including many using “realistic cartoony” and/or “superheroic” styles. *Realistic* styles attempted to maintain proportions of figures more characteristic of real figures, without exaggerated qualities of faces or bodies. Finally, *Alternative* styles characterized drawings with unorthodox or otherwise unclassifiable representations. A breakdown of the quantity of comics from each of these styles in each global region are depicted in Fig. 3b.

We emphasize again that, though we provide these stylistic classifications, we do not consider them as homogenous. Our computational methodology allows for style to be considered a continuous dimension, as reflected in the clustering of datapoints for comics Fig. 3a, allowing additional subdivisions and/or confirmation by other methods of categorization.

### Language

The languages of the comics were classified based on various factors. We first assessed the language represented within the text of the comics themselves, and if comics were translated, the final language assignment used the original, non-translated language. If comics had no text at all (13% of the corpus, 140/1030), we assigned languages based on the dominant language spoken in the country of origin. Based on this assessment, the corpus consisted of 55 spoken languages from 26 language families.

### Areas of analysis

**Fig. 4:**
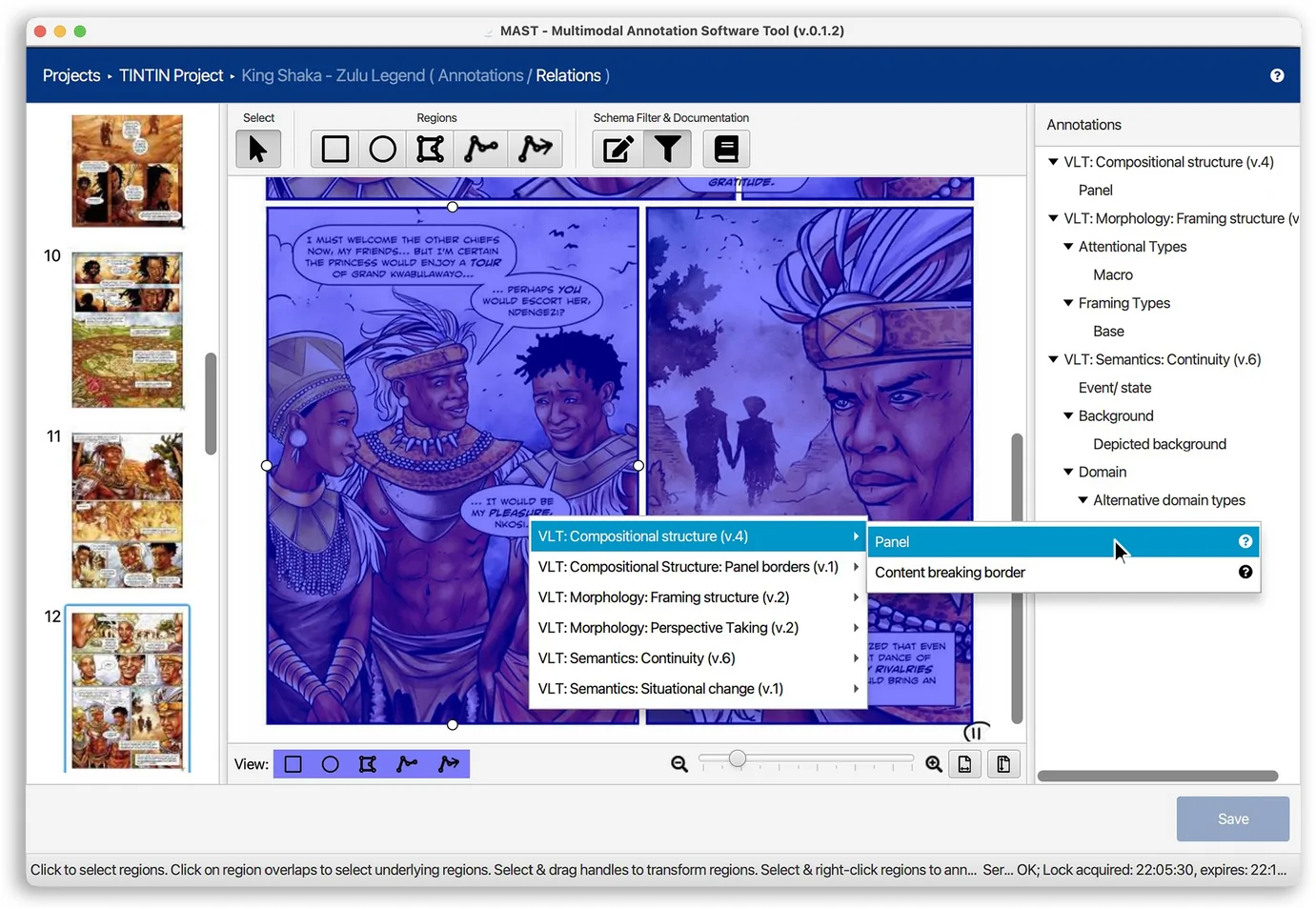
A screenshot from the MAST interface showing the annotation of a panel in *King Shaka: Zulu Legend* by Luke Molver (2019, Story Press Africa, South Africa)

All comics in the TINTIN Corpus were annotated using the Multimodal Annotation Software Tool, or MAST (Cardoso & Cohn, 2022). This software allows for the selection of vector-based regions along the surface of a visual representation, and then these regions are able to be annotated with user-based annotation schemes, as in Fig. 4. For this study, we used the “Panel” annotation in the Visual Language Theory: Compositional Structure v.4 annotation schema (Cohn et al., 2024).

Across all 1,030 comics from 144 countries or territories, this amounted to a total of 76,199 panels across the whole corpus within 14,324 pages. Here, we consider “pages” as a coherent *canvas* of the comic, whether as the space of an individual page canvas, or as a spread constituting multiple “pages.” MAST’s interface does not distinguish individual pages within a multi-page canvas, and thus our analysis considers two-page spreads as single “pages” (i.e., a single canvas) though they may have constituted two pages, in print form. We only considered multi-page spreads as individual pages if they constituted a coherent composition across a single canvas. Annotators independently selected panel regions, annotated them as “panels”, and then provided a number for their order in the page in the annotation notes field.

The whole TINTIN Corpus was annotated by 56 trained annotators implementing a variety of annotation schemes (Cohn et al., 2024), within which panels were annotated by 21 annotators. We used a multi-stage process where annotators were first trained in the theory and procedures of the annotation schemes, then annotated practice comics which were discussed. Only when practice annotations were deemed sufficient did annotators progress to the full corpus. In general, panel annotations receive high agreement (Edlin & Reiss, 2021), but all panel annotations in the corpus were checked multiple times by a supervisor and at least one other annotator, and questionable annotations were then discussed and/or altered. As panels formed the basis for various other annotations (Cohn et al., 2024), panels were additionally implicitly checked at least 10 to 15 more times throughout the annotation process of the whole corpus.

We considered “panels” as a unitized expression of a visual narrative sequence. A canonical panel has a definable border that encapsulates its graphic and/or textual content, but we also identified panels as units that had no borders, had content spilling out of their borders, had content extending to the edge of a page (bleed), overlapped in their content with other panels (such as in collages), or were representations of figures alone (such as a character that overlaps several panels, where the character is themselves a panel). While many panels are distinguished by their content as storytelling units, panels can also be devoid of pictorial and/or textual content as “null” or “empty” panels. In addition, content could extend across panels, such as in “divisional” panels which use multiple panels spanning across a single picture (Cohn, 2007, 2024). While such relationships with content were considered in the annotation procedures, in this study, we do not here address these interactions related to their “paneling structure” (Cohn, 2024) or borders, which were annotated in other annotation schemes, and which are available within the whole TINTIN Corpus. Rather, here we just investigate aspects of the regions annotated as panels alone.

### Language typology

To assess typological properties of morphological complexity found in spoken languages we use two different corpora of language data. First, we used the data for “word structure” from Koplenig et al. (2017) which provides a graded measure of complexity for word forms. Second we use the record of “Inflectional synthesis of the verb” (Bickel & Nichols, 2013) from the World Atlas of Language Structures (WALS), which details how many inflectional categories (e.g., agreement, tense/aspect/mood, evidentials, etc.) are inflected on verbs in a language. WALS records these as bigram categories which were then averaged and used numerically (i.e., “4 to 5 categories” was averaged to 4.5, etc.). As not all languages present in the TINTIN Corpus were included within these corpora, only a subsection of the corpus was included in these analyses. The Word Structure measure included 946 (92.8%) comics, and the WALS inflectional categories covered 764 (74.2%) comics.

### Data analysis

MAST outputs data of two primary types. First, it provides data for each instance of a given annotation, allowing us to assess the frequency of different phenomena in the corpus. We here analyze this annotation data by calculating how many panels per page appear on each page of a book. A second type of data comes from the graphical aspects of MAST’s selection of regions for annotations, yielding information about the size of a given region associated with an annotation. MAST does record data about absolute sizes of pages, but because comic pages in our corpus may differ in their size given both the digital dimensions and resolution (whether scanned or originally digitally created), absolute size is less informative for our analyses. Instead, we here report the relative size of regions annotated as panels to the size of the page. Because of this, a panel region would only occupy 100% of a page if it extended to the edge of the page (a “full bleed”), and otherwise most panels were separated by gutter space.

We begin with analyzing basic descriptive statistics for the mean panels per page and relative size of panels across the whole corpus. Here we ask whether the average number of panels and size of panels vary across the various dimensions of region, style, or language typology (word structure score and verb inflection score). This analysis used repeated measures ANOVAs with the dependent variable calculated for each page in a document, and pages modeled as repeated measures from a document. As such, the full model for all analyses was lmer(measure ~ style + region + wordStructure + verbInflection + year + (1 | documentID)). Models were constructed and compared using the lmer package (version 1.1–35.2) in R (version 4.3.2). Style, region, document ID, and year were coded as categorical variables while both dependent measures, word structure, and verb inflection were continuous variables. Post-hoc comparisons were conducted using the emmeans package (version 1.10.5) using the Tukey adjustment for multiple comparisons. R^2^ values were determined using the MuMIn package (version 1.48.1).

To additionally analyze the relationship between average size of panels per page and number of panels per page, a repeated measures analysis was performed. For these analyses the base 10 log of both values were used first in a model comparing panel count and size, lmer(logPanelCount ~ 1 + (1 | DocumentID)), and then including style, region, and year of publication date as fixed effects, lmer(logPanelCount ~ style + region + year + (1 | documentID)). Each comic (document) was included as a random effect.

## Results

### Descriptive statistics

**Fig. 5:**
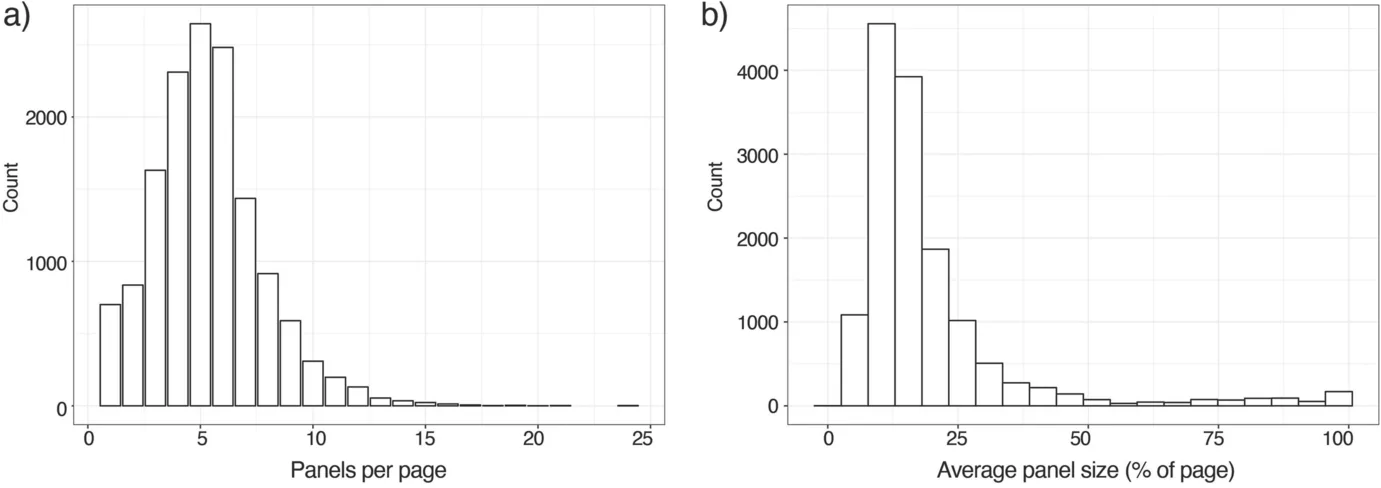
The distribution of **a** number of panels per page across comics of the TINTIN Corpus, and **b** the distribution of average sizes of panels

Across all 1,030 comics, we analyzed 14,324 pages which overall averaged 13.91 (median 13) pages per book. The distribution of the number of panels is reasonably normal with a right skew (Fig. 5a), with an overall average of 5.32 panels per page, and a median of 5 panels (SD = 2.4). The average relative panel size correspondingly had a mean size of 19.45% of the page (median = 14.57%, SD = 16.5%). Here the distribution of panels sizes across the corpus also had a reasonably normal distribution but also with a right skew (Fig. 5b).

### Panels per page

We next examined the variables involved in the number of panels per page by comparing models with style, global region, publication date, and language typology measures (word structure, verb inflection). Model comparison indicated that the full model best accounted for the data, and we thus report on each of the follow up analyses.

### Region and style

**Fig. 6:**
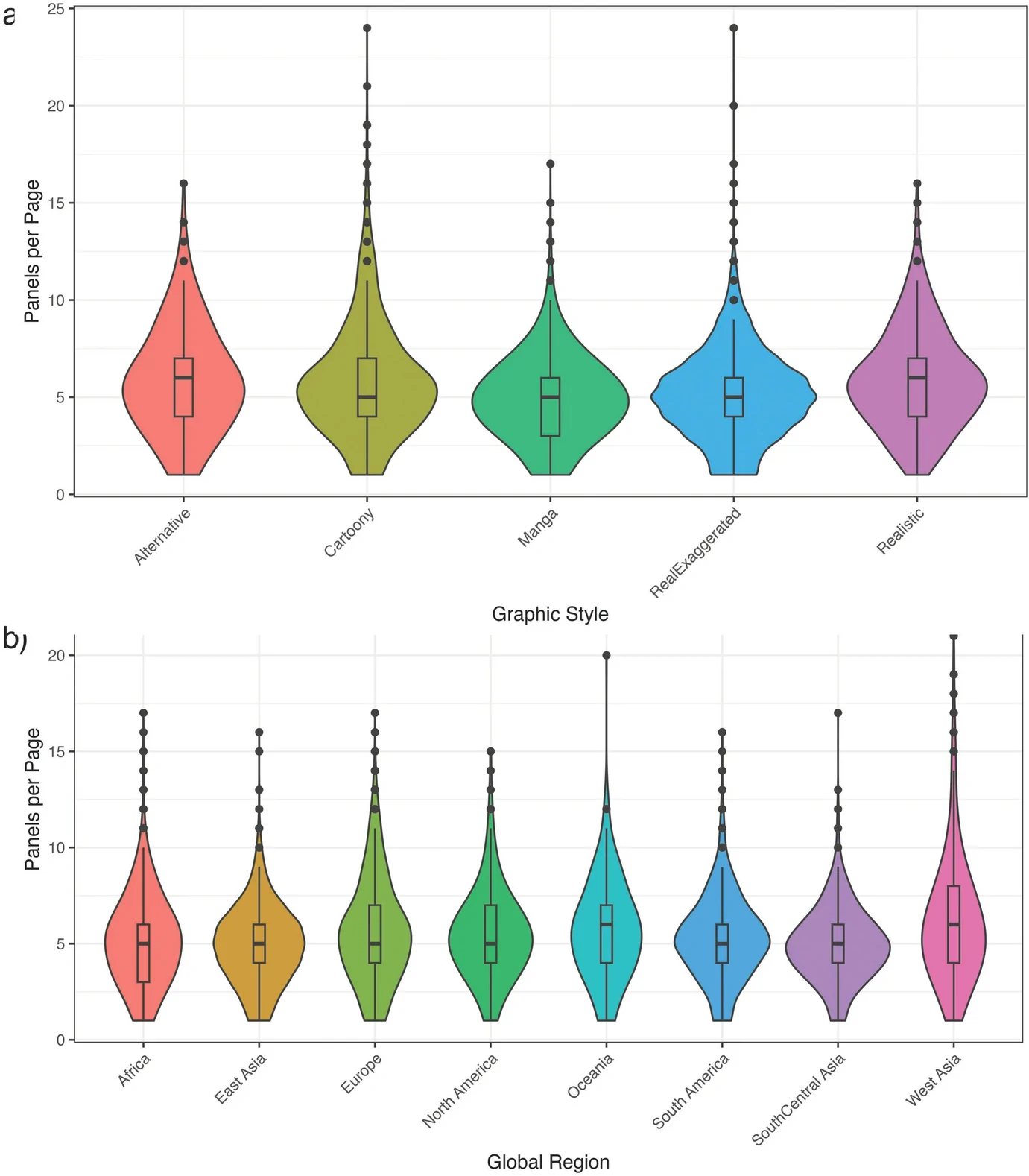
Average numbers of panels per page in comics **a** with different styles, and **b** from different global regions

A repeated measures ANOVA indicated a main effect of global region on the number of panels per page, χ^2^(7) = 24.2, p = .0011. Post-hoc pairwise comparisons of the estimated marginal means showed that this was most strongly motivated by panels from West Asia being greater than those in South America, t(638.99) = 3.42, p = .015, and South Central Asia, t(629.55) = 3.11, p = .041, as in Fig. 6b. All other pairwise comparisons were not significant at the 0.05 level. We also observed a main effect of style, χ^2^(4) = 19.9, p < .0001. Here Manga styled comics had fewer panels per page than those from Cartoony styles, t(638.99) = 3.42, p = .015, but we found no other significant comparisons (Fig. 6a). It is worth noting though that the estimated marginal mean difference between Manga and Realistic (0.77) styles was nearly identical to the difference between Cartoony and Manga (0.77). However, the standard error of the Manga—Realistic difference (0.31) was much higher than Cartoony—Manga (0.21) difference, resulting in only a marginal difference (p = .089) likely due to the smaller sample size of Realistic styled comics.

**Fig. 7:**
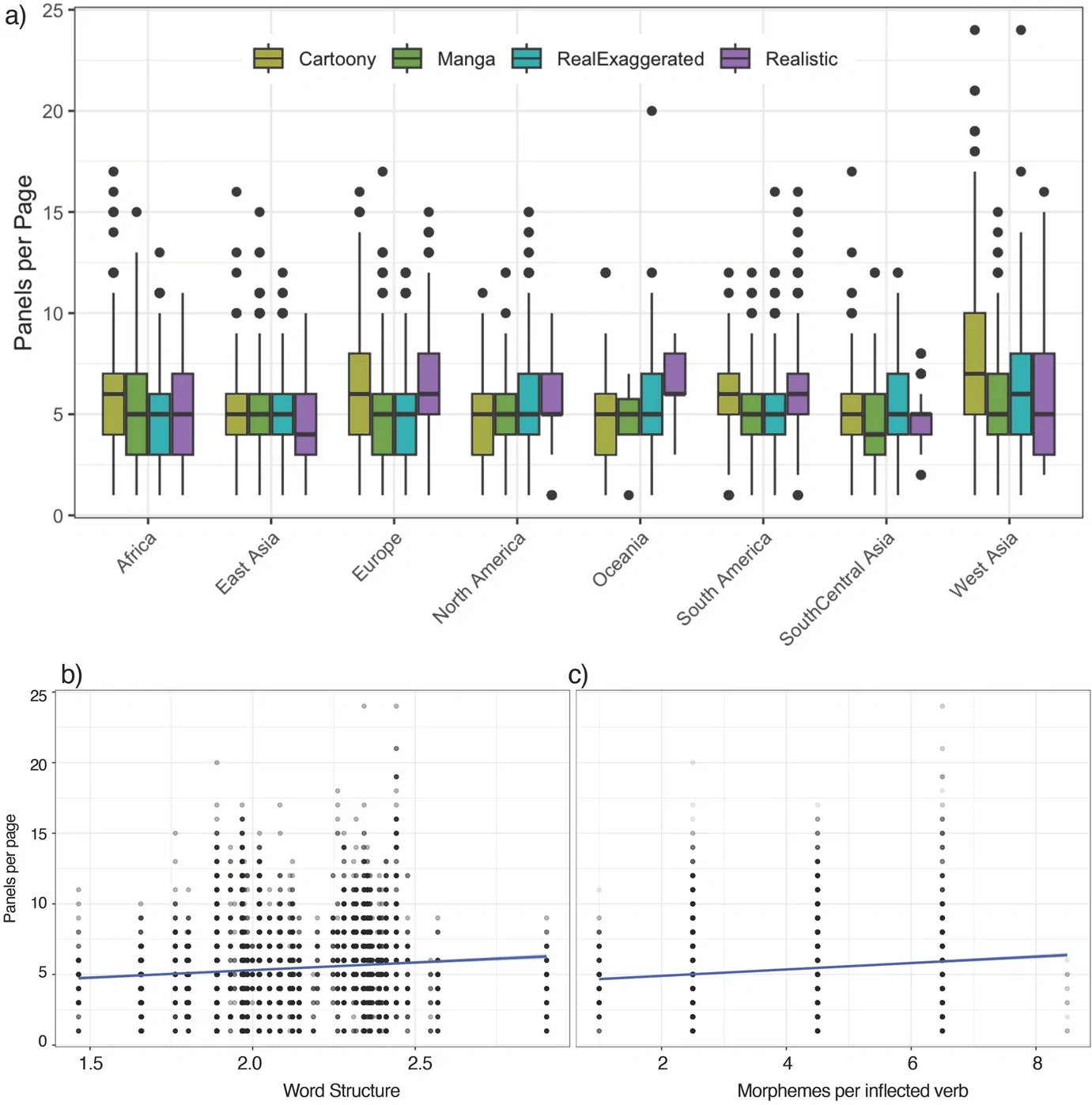
Average panels per page **a** across both global region and styles, and predicted by two measures of the complexity of words, **b** word structure (Koplenig et al., 2017) and **c** the number of morphemes inflected on verbs (Bickel & Nichols, 2013)

We followed up these main effects by examining an interaction between style and global region (Fig. 7a), which was also significant, χ^2^(21) = 50.17, p < .0001. We here excluded the Alternative style due to it not appearing in all regions. Post-hoc pairwise comparisons of the estimated marginal means between styles for each region (48 total) again show a difference between Cartoony and Manga styles in the West Asia region, t(634) = 5.84, p < .0001, but no other significant differences.

### Language typology

Our analysis of language typological variables showed a “trending” but not significant main effect of word structure (Koplenig et al., 2017) on the number of panels per page, χ^2^(1) = 3.22, p = .07, and a significant effect of the number of morphemes inflected on verbs (Bickel & Nichols, 2013), χ^2^(1) = 16.9, p < .0001. These results arose because greater numbers of panels per page appeared in comics produced by authors from languages that had more complexity per word (Fig. 7b) or morphemes inflected per verb (Fig. 7c).

### Publication date

We also observed a significant effect of the year of publication date on the number of panels per page, χ^2^(67) = 139.4, p < .0001. Post-hoc pairwise comparisons of the estimated marginal mean differences between years show a significant difference between 1946 and other years, including 1994, 2012, 2013, and 2015 (p < .05). All other comparisons were not significant at the 0.05 level.

### Size of panels per page

We next analyzed the size of panels relative to their pages, again using models with style, global region, publication date, and language typology measures (word structure, verb inflection). Here we found no main effect of global region on the size of panels χ^2^(7) = 10.97, p = .14, and though we found a main effect of style, χ^2^(4) = 10.7, p = .030, no post-hoc pairwise comparisons yielded significant differences. Language typology measures did reveal effects on size of panels, with significant main effects arising for both word structure scores, χ^2^(1) = 5.40, p = .020, and number of morphemes inflected on verbs, χ^2^(1) = 9.13, p = .002. These analyses revealed that as the languages of the comics’ word forms were more typologically complex, they used smaller panels. There was not a significant effect of publication date on size of panels per page, χ^2^(67) = 62.08, p = .65.

### Panel size x frequency

To evaluate the relationship between average size of panels per page and number of panels per page, we performed a repeated measures analysis with the base 10 log of both values. When compared with a null model containing only random effects, we found a significant main effect of the log panel size on the log of panel count, χ^2^(1) = 35,610, p < .0001. The log of panel size measure explained 89.3% of the variance (i.e., marginal R^2^ = 0.893) in the log of the panel count alone. When included with style, region, and year the percentage of variance explained increases to 89.6% (marginal R^2^ = 0.896), and the significant main effect of the log of panel count remained, χ^2^(1) = 35,546, p < .0001. The full model, including document as a random effect, explains 95.3% of the variance (R^2^ = 0.953).

**Fig. 8:**
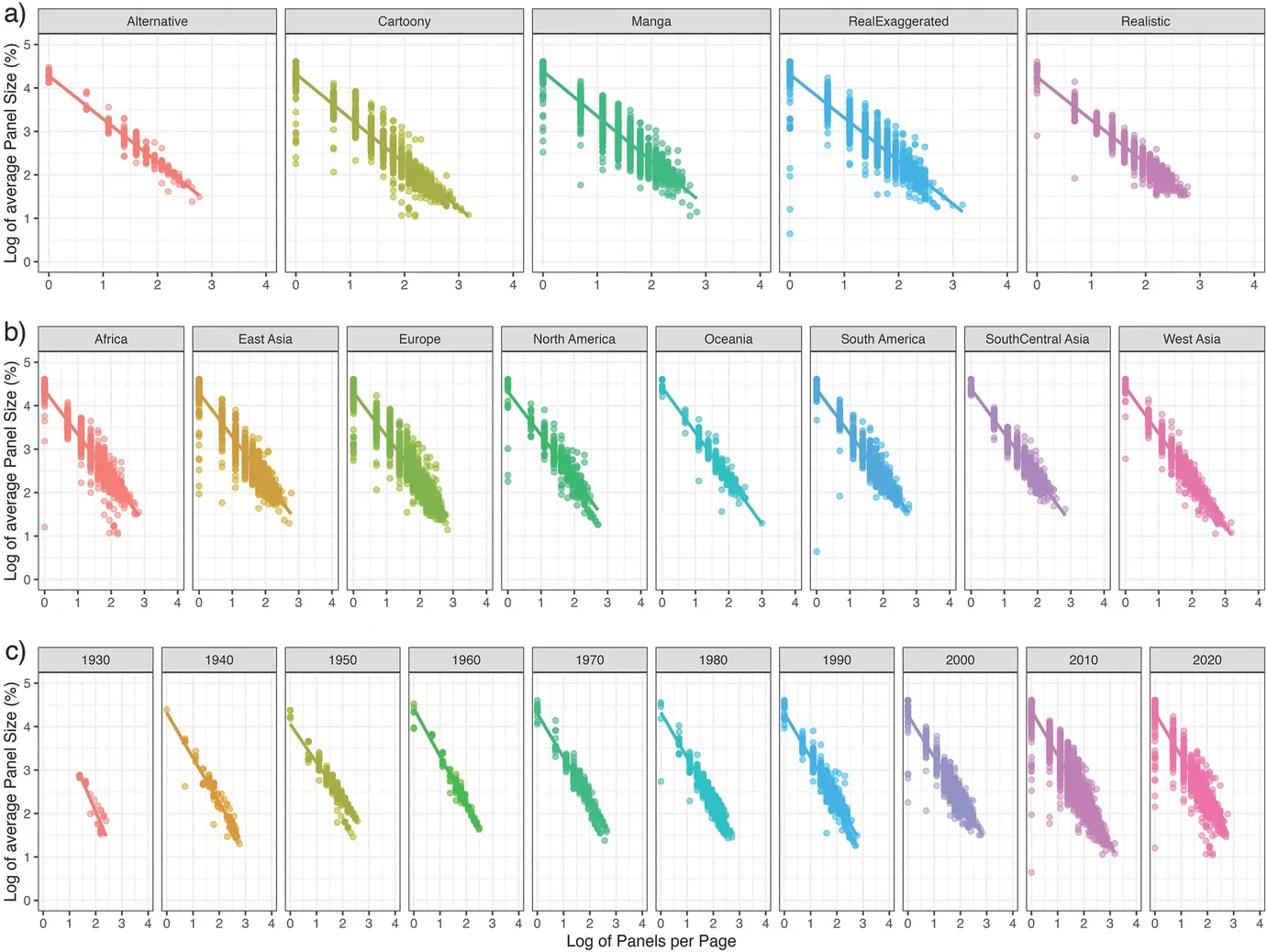
The relationship between average panels per page and relative panel size (log-transformed) across **a** comic styles, **b** their global regions, and **c** publication dates

These analyses revealed a tradeoff where fewer panels per page were larger and more panels per page were smaller. This tradeoff persisted across all dimensions of analysis, remaining the same in all styles (Fig. 8a), regions (Fig. 8b), and years of publication dates (Fig. 8c).

## Discussion

Here we analyzed 76,199 panels from 1,030 comics across 144 countries to investigate the variability and consistency across frequency and size of comic panels. Overall, we found that the frequency of panels per page varied across several dimensions including the region of the world the comics came from, their style, and their publication date. Nevertheless, we also observed a consistent trade-off between the frequency of panels per page and the size of panels, and this distribution was consistent across these dimensions of variability. Together, these results suggest that panels per page in comics involve a tension between aspects of diversity and universality similar to language and other expressive systems.

Overall, we observed an average of 5.32 panels per page with a median of 5 panels per page. This amount is slightly lower than the overall averages observed in the VLRC, where pages had an average of 6.4 panels per page (Cohn, 2024). This average is more consistent with prior observations of VLRC panel counts in pages from Asian and North American comics rather than the higher panel counts from European comics. In the TINTIN Corpus, the styles of Manga had the least number of panels per page, though this only differed statistically from Cartoony styled comics, which had the most panels per page, and was most pronounced in comics from West Asia. One possible reason for this similarity between the overall average number of panels per page here with only Asian and American comics in the VLRC may be the greater proportions of Manga and “Real-Exaggerated” styles in the TINTIN Corpus. In addition, over 80% of the TINTIN Corpus was published after 2000, while only 47% of the VLRC was published after 2000, and there it was observed that the number of panels per page decreased over time (Cohn, 2024). Here too we observed an effect of publication date. This overall finding of an interaction between global region and styles in our results contrasts with findings in the TINTIN Corpus for other structures that styles were a better predictor than, or maintained consistent structures across, global regions (Cohn et al., 2025a, 2025b, 2026). Along with publication date, this finding of an effect of global region may arise due to influence of the comics’ formatting. Prior work has emphasized that the number of panels per page may be influenced by the physical size of pages themselves (Lefèvre, 2000). For example, the larger physical size of Franco-Belgian albums may contribute to greater numbers of panels per page in European comics in the VLRC (Cohn, 2024), and in visual inspection of TINTIN Corpus data, relative to the size of manga or other comics. While such formatting may indeed exert an influence, the digital formatting of the TINTIN Corpus makes it difficult to evenly assess such absolute physical properties. In addition, some factors may have led to less constraint on publication format. First, many of these comics were created to be digital, with no physical constraints on sizing (though with possible constraints on intended browser presentation). Second, amateur comics in the corpus may not have been meant for printed distribution. Analysis of the “format” of the works (i.e., webcomic vs. album vs. pamphlet) may be informative to further explore this potential of variance and/or its interaction with styles and global regions.

Because the TINTIN Corpus also records information about the spatial properties of pages, our analysis also assessed the relative sizing of panels on pages. Here, we observed that panels occupied an average relative size of 19.45% of the page area with a median of 14.57%. This is consistent with the observation of there being an average frequency of roughly 5 panels per page, with panels occupying slightly less than one-fifth to one-sixth of the area of a page. While we did not explicitly examine aspects of page layout composition here, that grids have been observed as the most prevalent layout (Cohn, 2024) would imply that a prevailing default layout may be a 2 × 3 or 3 × 2 panel grid, which additional analysis of layout in the corpus could confirm.

Interestingly, panels per page and panel size were skewed differently in their distributions. While both displayed a right skew (Fig. 5), the numbers of panels per page appeared to have a slightly more normal distribution than panel sizes. This suggests that while the numbers of panels may vary, their sizes more consistently occupy between 10 to 20% of the page. Such consistency may imply there is an “optimal” panel size that authors have arrived at, whereby variation is rendered either larger or smaller relative to this prototype.

One curious finding is that, though we found that style, region, and language typology affected the numbers of panels per page, we largely did not find such effects on differences between the sizes of panels. This also comes despite a strong negative relationship between panels per page and their size (discussed below), which would imply that panel sizes would have an inverted relationship to that of panels per page (i.e., for comic types with more panels per page, we would expect similar effects of larger panels). This is likely due to the difference in variance between these measures, again in connection with the observed skewness discussed above.

In analyses of both the frequency of panels and their change over time, we observed a relationship between frequency and size of panels that resembled a trade-off: more panels per page were associated with smaller panels, and fewer panels per page were associated with larger panels. This result seems intuitive, since a finite canvas entirely filled with panels will mean that more panels will naturally become smaller. However, there are potentially an infinite number of possible layouts, including many that would not fill the entire space of a canvas. So, though this negative relationship between size and frequency confirms the logical expectation, it was not inevitable.

Overall, this relationship between frequency and size is consistent with the Menzerath–Altmann Law (Altmann, 1980; Menzerath, 1928), a “linguistic law” specifying that the larger a construct, the smaller the size of its constituents. While we had no absolute measure of page sizes, the tradeoff in frequency and size here resembles that observed in the Menzerath–Altmann Law, where here larger segmental units (panels) are associated with fewer units. Consistent with this being a universal property, we observed that this distributional trade-off persists across all dimensions where panels otherwise varied, including global regions, styles, and publication dates (Fig. 8a–c).

Finally, language typology also showed a relationship with both panel count and size. Drawing on data from two linguistic corpora (Bickel & Nichols, 2013; Koplenig et al., 2017), we observed that languages with more complex words were associated with more panels per page. This association correspondingly led to languages with typologically more complex words to also have smaller panels, in line with the tradeoff between number of panels and size. Such results imply a possible “permeable” influence of complex units in one modality (speech) being reflected in that of another modality (graphics). However, it is worth stressing that this comparison is not a direct analogy, as whole pages are not comparable as sequential units in visual narrative like words are in sentences. There is also considerably greater complexity within panels as sub-units than within the morphemes of words. Thus, if there is any validity to such an influence, it must operate at a fairly general level. This finding should therefore be taken with caution and additional research will be required to tease out its implications.

Altogether, these data show that the number and size of panels per pages in comics are influenced by a number of factors, yet demonstrate some fairly consistent—if not universal—tendencies. This work has been made possible through the TINTIN Corpus, one of the largest and most diverse corpora of comics yet compiled, and which has the promise to provide additional insights across various other dimensions of structure of graphics and their relationship to language and cognition.

## Data Availability

All annotation data along with vector-based recreations of all page layouts of the corpus can be found in its DataverseNL repository: https://doi.org/10.34894/KSVSRL.
